# Salirasib Inhibits the Expression of Genes Involved in Fibrosis in Fibroblasts of Systemic Sclerosis Patients

**DOI:** 10.1002/iid3.70063

**Published:** 2024-11-27

**Authors:** Mina Sadeghi Shaker, Mohsen Rokni, Hoda Kavosi, Samaneh Enayati, Elham Madreseh, Mahdi Mahmoudi, Elham Farhadi, Mohammad Vodjgani

**Affiliations:** ^1^ Department of Immunology, School of Medicine Tehran University of Medical Sciences Tehran Iran; ^2^ Rheumatology Research Center Tehran University of Medical Sciences Tehran Iran; ^3^ Department of Immunology University of social Welfare and Rehabilitation Sciences Tehran Iran; ^4^ Research Center for Chronic Inflammatory Diseases Tehran University of Medical Sciences Tehran Iran; ^5^ Department of Epidemiology and Biostatistics, School of Public Health Tehran University of Medical Sciences Tehran Iran

**Keywords:** dermal fibroblast, fibrosis, salirasib, systemic sclerosis, TGF‐β1

## Abstract

**Background:**

Fibrosis is a principal sign of systemic sclerosis (SSc) which can affect several organs including the lung, heart, and dermis. Dermal fibroblasts of SSc patients are characterized by persistent and activated Ras and ERK1/2 signaling which stimulates extreme collagen and extracellular matrix synthesis. Salirasib is a Ras inhibitor that competitively prevents the adherence of GTP‐bound Ras to the plasma membrane, that inhibits Ras signaling. This study intended to clarify whether salirasib can influence fibrotic mediators in SSc fibroblasts.

**Materials and Methods:**

Dermal fibroblasts from 10 SSc patients were treated with salirasib in the presence of TGF‐β1, and mRNA levels of H‐Ras and genes related to fibrosis, such as *COL1A1, COL1A2*, *CTGF*, *TGF‐β1*, fibronectin, *ACTA2*, and *MMP1* was measured by real‐time PCR. The α‐SMA protein expression was analyzed by immunofluorescence staining.

**Results:**

In dermal fibroblasts of SSc patients, salirasib treatment, markedly downregulated the *H‐Ras* gene expression. In addition, the protein expression of α‐SMA and gene expression of *ACTA2* were inhibited upon salirasib treatment. Salirasib also significantly reduced the expression of *COL1A1*, and *COL1A2* genes and augmented the gene expression of *MMP1*. The mRNA levels of other genes related to fibrosis such as *FN1*, *CTGF*, and *TGF‐β1* were significantly decreased upon salirasib treatment.

**Conclusion:**

Considering salirasib significantly reduced the expression of genes related to the fibrosis process and α‐SMA gene and protein expression, and given significant upregulation of *MMP1* by salirasib, it can be considered as a new curative strategy for fibrotic diseases like SSc.

## Graphical abstract

Salirasib is a derivative of salicylic acid and has been known as an antagonist of the Ras protein molecule. To date, many studies have used salirasib as an inhibitor of the Ras protein molecule in various cancer cells, which has reduced the proliferation and migration of cancer cells. However, our study is the first to show the effect of salirasib on fibroblasts of patients with SSc. It was able to significantly reduce the factors involved in the process of fibrosis, which are known as Hall markers and the main cause of death in this disease. The inhibition of fibrosis by salirasib can be considered a potential therapeutic approach for these patients.

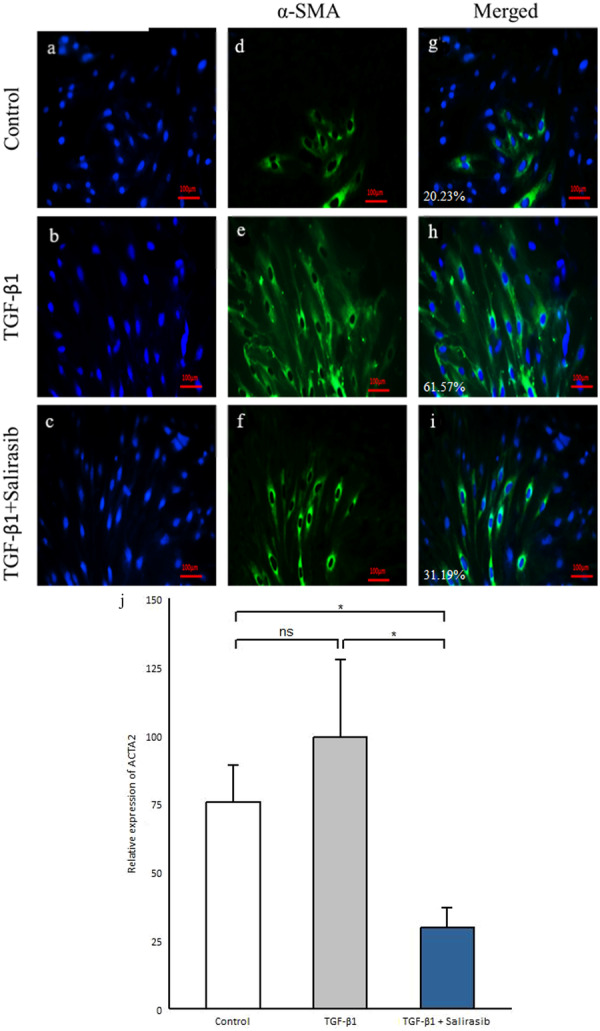

**Abbreviations**
α‐SMA: α‐smooth muscle actin
ACR: American College of Rheumatology
CTGF: connective tissue growth factor
DAPI: 4′, 6‐diamidino‐2‐phenylindole
dcSSc: diffuse cutaneous systemic sclerosis
DMEM: Dulbecco's Modified Eagle's medium
DMSO: dimethyl sulfoxide
ECM: extracellular matrix
ET‐1: endothelin‐1
FBS: fetal bovine serum
FITC: fluorescein isothiocyanate
FN1: fibronectin1
FSP: fibroblast surface protein
GAPDH: glyceraldehyde‐3‐phosphate dehydrogenase
IL‐6: interleukin‐6
lcSSc: limited cutaneous systemic sclerosis
MMP: matrix metalloproteinases
MTT: 3‐(4,5‐Dimethylthiazol‐2‐yl)‐2,5‐diphenyltetrazolium bromide
PBS: phosphate‐buffered saline
PDGF: platelet‐derived growth factor
ROS: reactive oxygen species
SSc: systemic sclerosis
TGF‐β: transforming growth factor‐bet
TUMS: Tehran University of Medical Sciences

## 1 Introduction

Systemic sclerosis (SSc) is an autoimmune connective tissue disease that is characterized by three particular features: vasculopathy, inflammation and autoimmunity, and fibrosis [1]. Fibrosis, a pathological hallmark of SSc, is a leading contributor to numerous mortality in SSc [2]. The fibrosis process is created through the extreme deposition of extracellular matrix (ECM) proteins including collagen and fibronectin around inflamed or damaged tissue. Besides, inhibition of ECM decomposition due to defects in the function and expression of matrix metalloproteinases (MMPs) especially MMP1 (collagenase‐1) may debilitate the physiological performance of the affected tissue and finally result in organ dysfunction [1, 3, 4]. Overproduction of ECM is predominantly orchestrated by fibroblasts [3]. Fibroblasts in the presence of transforming growth factor‐beta (TGF‐β) can be activated and differentiated into myofibroblasts [5]. Myofibroblasts are activated fibroblasts that are recognized by the α‐smooth muscle actin (α‐SMA) expression [6]. The excessive accumulation of myofibroblasts in connective tissues is account for the uncontrolled and excessive secretion of ECM during the appearance and development of fibrotic diseases, such as SSc [6, 7]. TGF‐β1 signaling takes place via two distinct pathways: the canonical pathway (Smad‐dependent) and the noncanonical pathway that activates the Ras‐MAPK pathway. Various profibrotic growth factors and cytokines, such as endothelin‐1(ET‐1), platelet‐derived growth factor (PDGF), and interleukin‐6 (IL‐6), have been reported in the serum and skin of SSc patients that recruit the Ras signaling pathway, resulting in fibroblast proliferation and survival, increased collagen type I/III and ECM synthesis, and decreased expression of MMP‐1 [8, 9, 10].

The Ras signaling pathway also has a crucial role in the trans‐differentiation of various cells such as pericytes, endothelial cells, epithelial cells, and fibrocytes into myofibroblasts [8]. Furthermore, Ras‐ERK1/2 signaling leads to augmented production of ROS in SSc fibroblasts [8]. Various factors such as TGF‐β1, PDGF, and anti‐PDGFR antibodies through fibroblast stimulation of SSc patients result in activating of Ras‐ERK1/2 signaling cascade that is followed by increased expression of reactive oxygen species (ROS). In a positive feedback loop, ROS increases the H‐Ras expression and activates ERK1/2 signaling, as well as causes the TGF‐β1 secretion, which has a main role in the fibrosis process [8, 11].

Salirasib is a Ras farnesyl cysteine mimetic that detach all active Ras isoforms from the plasma membrane and blocks downstream signaling [12]. According to previous studies, salirasib prevents migration, incursion, and cell multiplication in Ras‐mutated cancer cell lines [13, 14, 15] and is recently undergoing trials in patients with non‐small cell lung, and pancreatic cancers [16]. However, concerning the presence of the Ras signaling in non‐canoical pathway located in downstream of TGF‐β1 receptor and other profibrotic mediators and its crucial role in the fibrosis process by induction of fibrotic mediators and myofibroblast differentiation, the effects of salirasib on fibrosis in SSc have not yet been inspected. This study intended to evaluate whether the salirasib treatment of SSc fibroblasts can inhibit myofibroblast differentiation and the expression of genes related to the fibrosis process and debilitate the profibrotic activation of TGF‐β1.

## 2 Materials and Methods

### 2.1 Patients

Skin biopsies were obtained from the dorsal forearms of 10 patients (8 females and 2 males) with SSc with an average age of 44.30 ± 16.5 years, their disease had been confirmed by the rheumatologist according to the standards of the American College of Rheumatology (ACR) [17]. Skin biopsies were taken from patients with SSc who were referred to the Rheumatology Clinic of Shariati Hospital, Tehran University of Medical Sciences (TUMS). All individuals had a diffuse form of systemic sclerosis and modified Rodnan skin score (MRSS) [18] higher than 20, disease duration less than 5 years, and not taking two drugs (cyclophosphamide and mycophenolate mofetil) for the last 6 months. Informed consent was obtained from all participants before the biopsy, and the study was approved by the ethics committee of Tehran University of Medical Sciences (Ethics code: IR. TUMS. MEDICINE. REC.1399.1156).

### 2.2 Fibroblast Isolation and Culture

Dermal fibroblasts were isolated from each biopsy specimen using the explant technique [19]. The samples were cut into 2 × 2 mm^2^ pieces and completely adhered to the bottom of each well of a 6‐well plate, allowing the efficient separation of fibroblasts. Fibroblasts were grown to confluence in Dulbecco's Modified Eagle's medium (DMEM; Gibco, USA) supplemented with 10% fetal bovine serum (FBS; Biosera, France), 100 U/mL penicillin, and 100 μg/mL streptomycin (Sigma‐Aldrich, USA) in at 37℃ incubator with 5% CO_2_. When the confluency reached 80%–90% fibroblasts were sub‐cultured to a T25 flask (SPL, Korea). After three passages the purity of fibroblasts was confirmed by immunofluorescence staining for fibroblast surface protein (FSP). Fourth to sixth passage fibroblasts were used for further experiments.

### 2.3 Treatment Conditions

In all experiments described here, after plating and improvement of the cell growth for 24 h, a culture medium supplemented with 10% FBS was picked up and replaced by a serum‐free culture medium. After 24 h of serum starvation, the fibroblasts underwent initial treatment with 10 ng of TGFβ1. Following a 48‐h period, 12.5 μm of sarilasib was added, and the incubation continued for an additional 24 h.

### 2.4 MTT Assay

The MTT [3‐(4,5‐Dimethylthiazol‐2‐yl)−2,5‐diphenyltetrazolium bromide] assay was used to measure the toxicity of various concentrations of salirasib in fibroblast cells. Fibroblast cells were seeded in 48‐well microtiter plates at a concentration of 1 × 10^4^ cells in each well. After 24 h, cells were placed in a serum‐free medium. They underwent a 48‐h pretreatment with 10 ng/mL TGFβ1, followed by adding the salirasib at concentrations of 12.5, 25, and 50 μM for 24 h. At the end of this incubation time, MTT solution (Sigma‐Aldrich, USA) in phosphate‐buffered saline (PBS) (5 mg/mL) was added. After incubation with MTT at 37℃, the cells were centrifuged at 1000 g for 10 min. The supernatant was carefully picked up, and dimethyl sulfoxide (DMSO; Sigma‐Aldrich, USA) was added to each well. Then, the absorbance was measured at 570 nm using an enzyme‐linked immunosorbent assay reader (Biotek‐ ELx800, USA).

### 2.5 RNA Extraction and cDNA Synthesis

Total RNA was extracted from dermal fibroblasts using the SinaPure‐RNA kit (SinaClonBioScience, Iran) according to the manufacturer's instructions. RNA was quantified using a NanoDrop spectrophotometer (Thermo Fisher Scientific, USA). Total RNA was processed by cDNA synthesis or stored at −70°C until use. From each sample, 500 ng of RNA was used to synthesize cDNA using the RT‐ROSET Recombinant Kit (ROJE Technologies, Iran) in a total volume of 20 μL in a thermocycler. cDNA samples were stored at −70°C before the target cDNA was amplified.

### 2.6 Quantitative Real‐Time PCR (qRT‐PCR)

To determine the mRNA level of target genes, 250 ng of cDNA was employed as a template for an RT‐qPCR reaction with the RealQ Plus 2× Master Mix Green (Ampliqon, Denmark) by the Applied Biosystems StepOnePlus Real‐Time PCR System (Foster City, CA, USA). To amplify the genes PCR program was as follows: an initial activation at 95°C for 15 min was followed by an augmentation in target sequence number of 40 cycles at 95°C for 15, and 60°C for 1 min. The experiment was performed in duplicate and the expression of every gene was normalized to the transcript level of glyceraldehyde‐3‐phosphate dehydrogenase (GAPDH) gene. The relative expression of target genes was computed by the comparative Ct method (2^−^^ΔΔCT^). Primer sequences for all genes are provided in Table 1.

**Table 1:** The sequence of primers used in real‐time PCR.

GAPDH‐F	5′‐ACACCCACTCCTCCACCTTTG‐3′
GAPDH‐R	5′‐TCCACCACCCTGTTGCTGTAG‐3′
HRAS‐F	5′‐ATGACGGAATATAAGCTGGTGGT‐3′
HRAS‐R	5′‐GGCACGTCTCCCCATCAATG‐3′
ACTA2‐F	5′‐CTATGAGGGCTATGCCTTGCC‐3′
ACTA2‐R	5′‐GCTCAGCAGTAGTAACGAAGGA‐3′
CTGF‐F	5′‐AAAAGTGCATCCGTACTCCCA‐3′
CTGF‐R	5′‐CCGTCGGTACATACTCCACAG‐3′
TGFB1‐F	5′‐CGACTACTACGCCAAGGA‐3′
TGFB1‐R	5′‐GAGAGCAACACGGGTTCA‐3′
FN1‐F	5′‐AGGAAGCCGAGGTTTTAACTG‐3′
FN1‐R	5′‐AGGACGCTCATAAGTGTCACC‐3′
COL1A1‐F	5′‐GAGGGCCAAGACGAAGACATC‐3′
COL1A1‐R	5′‐CAGATCACGTCATCGCACAAC‐3′
MMP1‐F	5′‐ATTACACGCCAGATTTGCCAAG‐3′
MMP1‐R	5′‐AGAGTTGTCCCGATGATCTCCC‐3′
COL1A2‐F	5′‐GTTGCTGCTTGCAGTAACCTT‐3′
COL1A2‐R	5′‐AGGGCCAAGTCCAACTCCTT‐3′

### 2.7 Immunofluorescence Staining of Fibroblast Cells

To evaluate the effect of salirasib on the activation and differentiation of fibroblasts into α‐SMA positive myofibroblasts, immunofluorescence staining of fibroblasts was performed using an anti‐alpha smooth muscle actin antibody (Abcam, Cambridge, UK). Fibroblast cells were seeded in 24‐well plates, after serum starvation, treated with 10 ng/mL TGF‐β1 for 48 h and then 12.5 μM salirasib for 24 h. After rinsing with PBS buffer, the fixation of fibroblast cells was accomplished with cold methanol for 5 min. The cells were washed with PBS buffer and incubated with PBS/Triton‐X100 buffer including 1% bovine serum albumin (BSA; Sigma‐Aldrich, USA) for 1 h. The cells were incubated with primary antibody for anti‐alpha smooth muscle actin (Gene Tex, North America). Then, Goat Anti‐Rabbit IgG Antibody (HexaBiogen, Morocco) conjugated fluorescein isothiocyanate (FITC) as a secondary antibody was added and incubated for 1 h at room temperature. Nuclei were stained with the fluorescent dye 4′, 6‐diamidino‐2‐phenylindole (DAPI; Sigma‐Aldrich, USA). Then, fibroblast cells were assessed using an inverted fluorescence microscope (Olympus, Japan). Quantitation of α‐sma fluorescence from the control group showed a median of 20.23%, while the median fluorescence in the TGF‐β1 treated group was 61.57% and that in the group treated with TGF‐β1 and salirasib was 31.19%.

### 2.8 Statistical Analysis

Data were expressed as mean ± SEM. After assessing the normality distribution of quantitative variables by Shapiro–Wilk test, the Spearman's correlation test was used to investigate the relationship between fibrotic genes at baseline. Also, the comparisons among three paired groups were performed using the Friedman test and if statistically significancy was appeared, the Wilcoxon test with Bonferroni adjustment was used to pairwise comparison. Pairwise comparisons of cell survival in MTT plot were done by Dunnett post hoc test. All analyses were carried out using R software, version 4.2.3 and *p* values less than 0.05 were considered statistically significant.

## 3 Results

### 3.1 *H‐Ras* Has a Significant Positive Correlation With Fibrotic Genes

**Figure 1:**
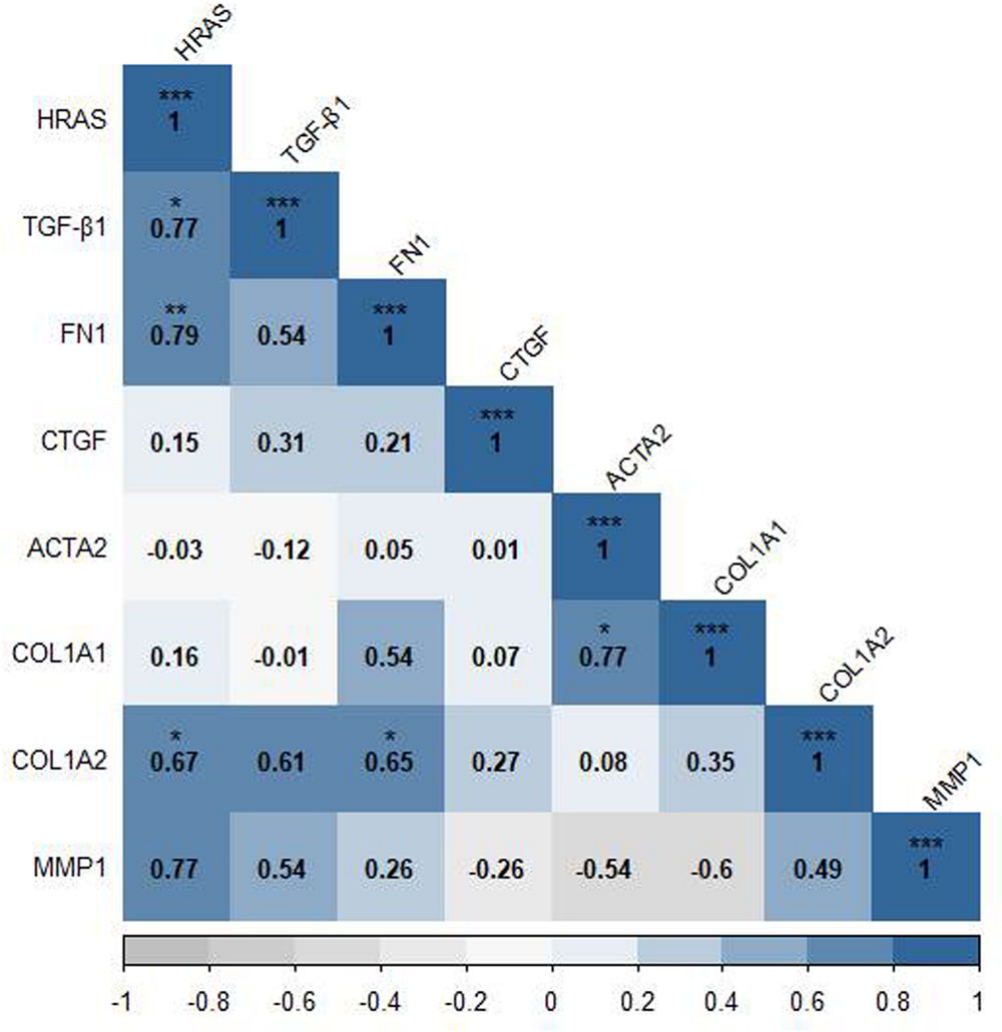
There is a significant positive correlation between the *H‐Ras* gene and fibrotic genes. spearman's correlation coefficient showed a significant positive correlation between the *H‐Ras* gene and *COL1A2*, *FN1*, and *TGF‐β* genes. Furthermore, *COL1A1* and *ACTA2* genes, as well as *COL1A2* and *FN1* genes were significantly correlated. *p < 0.05, **p < 0.01, ***p < 0.001.

To evaluate the relationship between the genes related to fibrosis, Spearman's correlation was used between the expression of genes involved in the process of fibrosis in the baseline state of SSc dermal fibroblasts. Spearman's correlation coefficient represented a significant positive correlation between the *H‐Ras* gene and *COL1A2*, *FN1* (Fibronectin 1), and *TGF‐β* genes (*r* = 0.67, *p* = 0.039; *r* = 0.79, *p* = 0.01; *r* = 0.77, *p* = 0.014 respectively). Besides, *COL1A1* and *ACTA2* genes (*r* = 0.77, *p* = 0.014), as well as *COL1A2* and *FN1* genes (*r* = 0.65, *p* = 0.049) were significantly correlated (Figure 1).

### 3.2 High Concentrations of Salirasib Are Toxic to Fibroblast Cells

**Figure 2:**
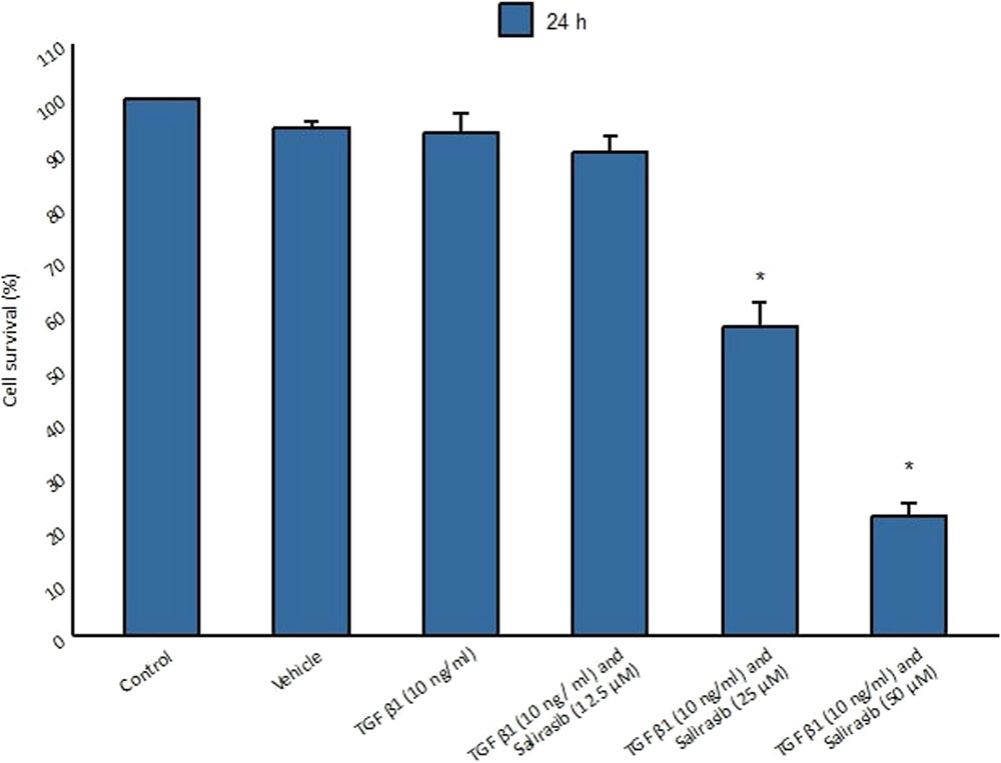
The effect of different concentrations of salirasib on the survival rate of fibroblast cells. The concentration of 12.5 μM salirasib does not affect the survival of fibroblast cells.

The MTT assay was used to assess the viability of fibroblasts treated with salirasib. For this purpose, fibroblast cells were cultured in a serum‐free medium and treated with cytokine TGF‐β1 for 48 h and then different concentrations of salirasib (12.5, 25, and 50 μM) for 24 h. The results showed that cells had the highest survival rate at 12.5 μM salirasib concentration whereas the cells treated with 25 and 50 μM salirasib showed the less survival rate compared to the control group (Figure 2).

### 3.3 Salirasib Represses *H‐Ras* Gene Expression in SSC Fibroblasts

**Figure 3:**
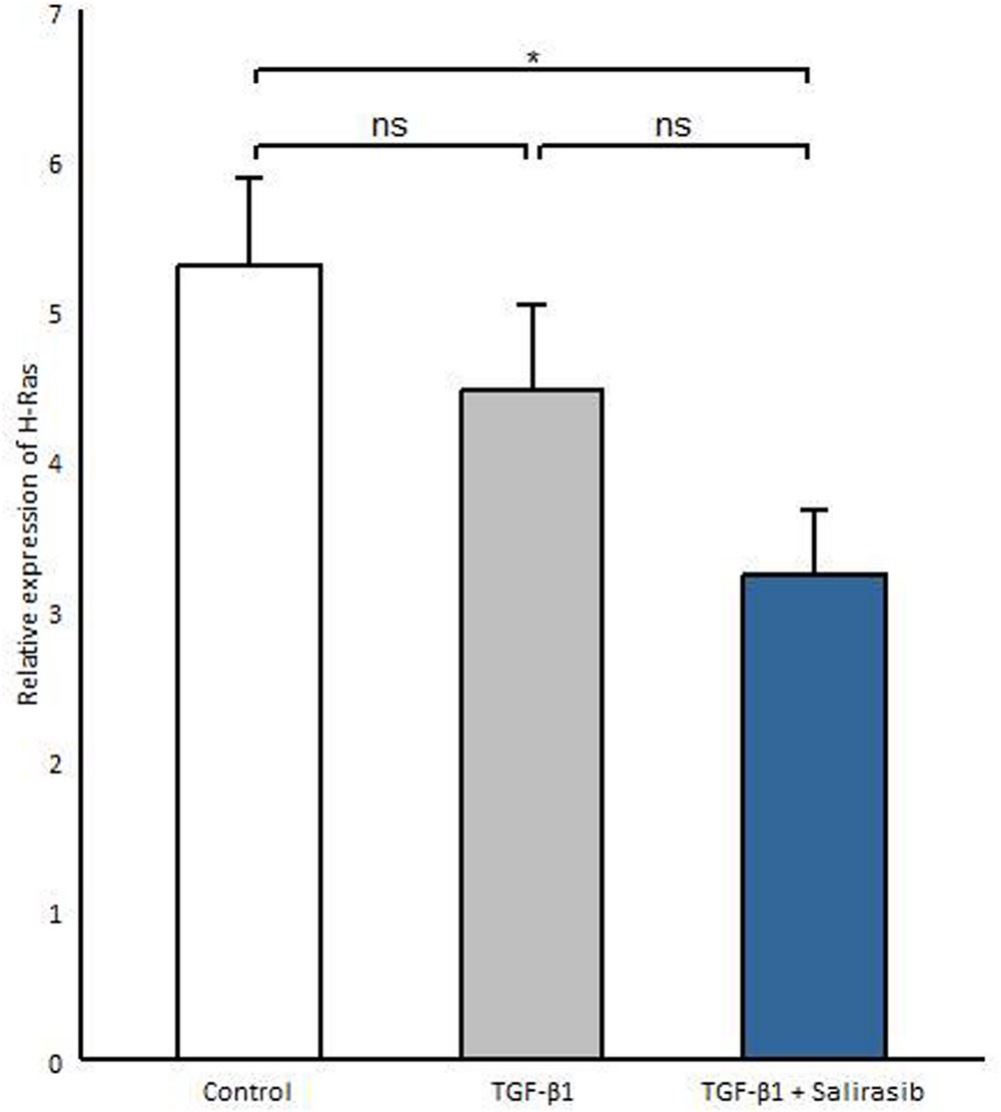
Effect of salirasib on *H‐Ras* gene expression in the presence of TGF‐β1 cytokine. The transcription levels of *H‐Ras* genes were analyzed after treatment of the patient's fibroblasts with 12.5 μm salirasib in the presence of 10 ng/ml TGF‐β1. Values shown are the mean ± SEM. *p < 0.05, **p < 0.01, ***p < 0.001.

Considering that salirasib inhibits Ras activity and function [14], we investigated whether salirasib also affects *H‐Ras* gene expression. Thus, we evaluated the efficacy of salirasib on *H‐Ras* mRNA expression in SSc fibroblasts. We observed that salirasib significantly reduces *H‐Ras* gene expression compared to the untreated group (*p* = 0.024) (Figure 3).

### 3.4 Salirasib Inhibits α‐SMA‐Positive Myofibroblast Differentiation

**Figure 4:**
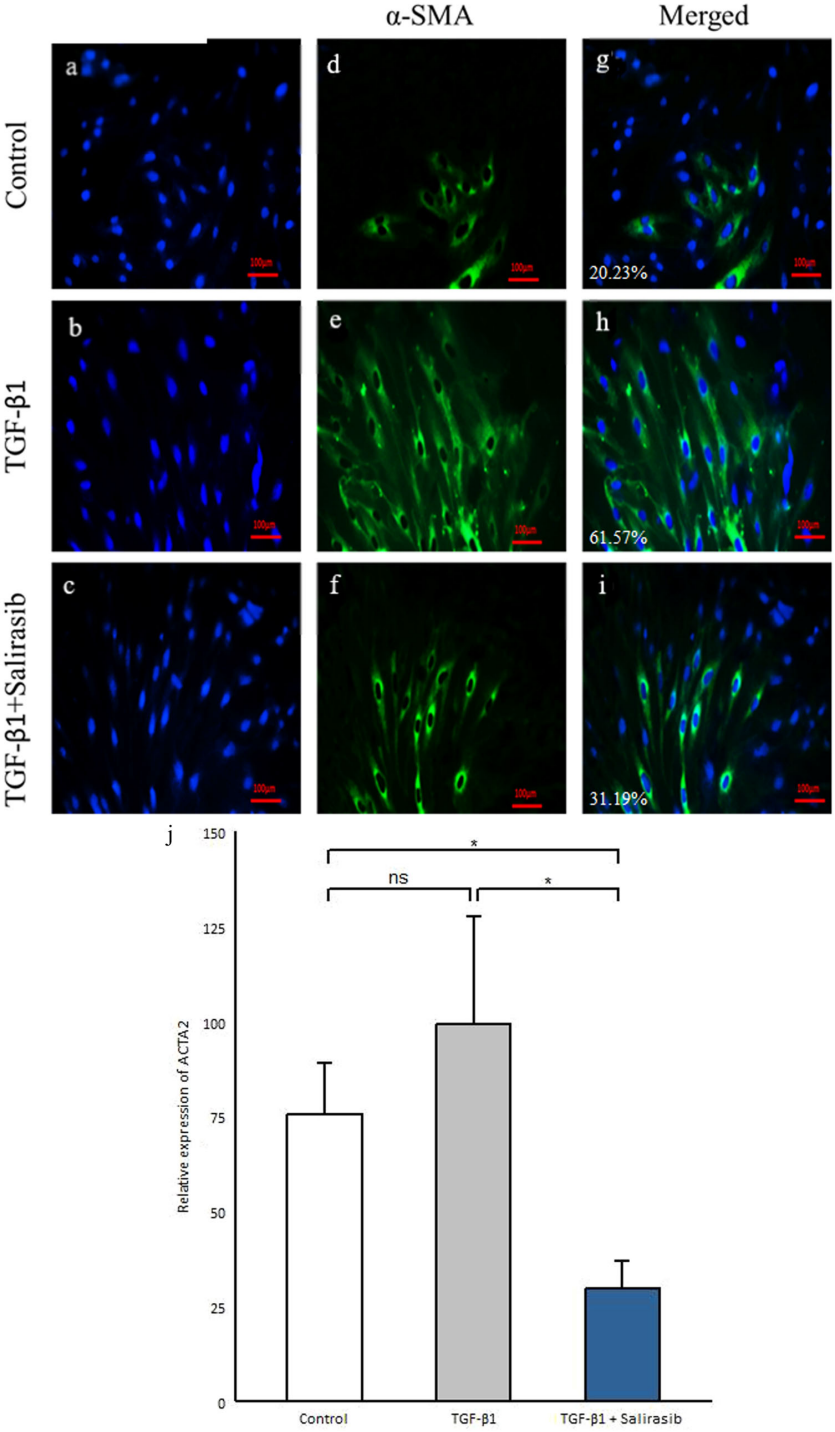
Effects of salirasib on the differentiation of fibroblasts into α‐SMA‐positive myofibroblasts in the presence of TGF‐β1. Immunofluorescence staining of alpha‐smooth muscle actin (myofibroblastic marker) with FITC‐labeled antibodies in fibroblasts cells from the control group (a) TGF‐ β1 treated group (b) the group treated with TGF‐β1 and salirasib (c), and nuclei with DAPI in fibroblasts cells from the control group (d) TGF‐β1 treated group (e) the group treated with TGF‐β1 and salirasib (f). Merged images of control group (g) TGF‐β1 treated group (h) the group treated with TGF‐β1 and salirasib (i) the expression of myofibroblastic marker mRNA level (*ACTA2*) using real‐time PCR (j). The median percentage fluorescence in the control group was 20.23%, in the TGF‐β1 treated group was 61.57%, and in the group treated with TGF‐β1 and salirasib was 31.19%. Values shown are the mean ± SEM. *p < 0.05, **p < 0.01, ***p < 0.001.

TGF‐β1 has a positive role in fibrosis and the differentiation of fibroblasts into α‐SMA‐positive myofibroblasts [3]. Our results showed that TGF‐β1 led to an enhancement in α‐SMA‐positive myofibroblasts (Figure 4e,h). To investigate the effect of salirasib on the α‐SMA‐positive myofibroblasts, we investigated the α‐SMA protein expression and its gene expression (*ACTA2*). The results showed that salirasib treatment of SSc fibroblasts leads to reduced differentiation of fibroblasts into α‐SMA‐positive myofibroblasts and decreased *ACTA2* gene expression (*p* = 0.012) (Figure 4f–j).

### 3.5 Salirasib Induces *Mmp1* Expression While Decreasing the Expression of Collagen

**Figure 5:**
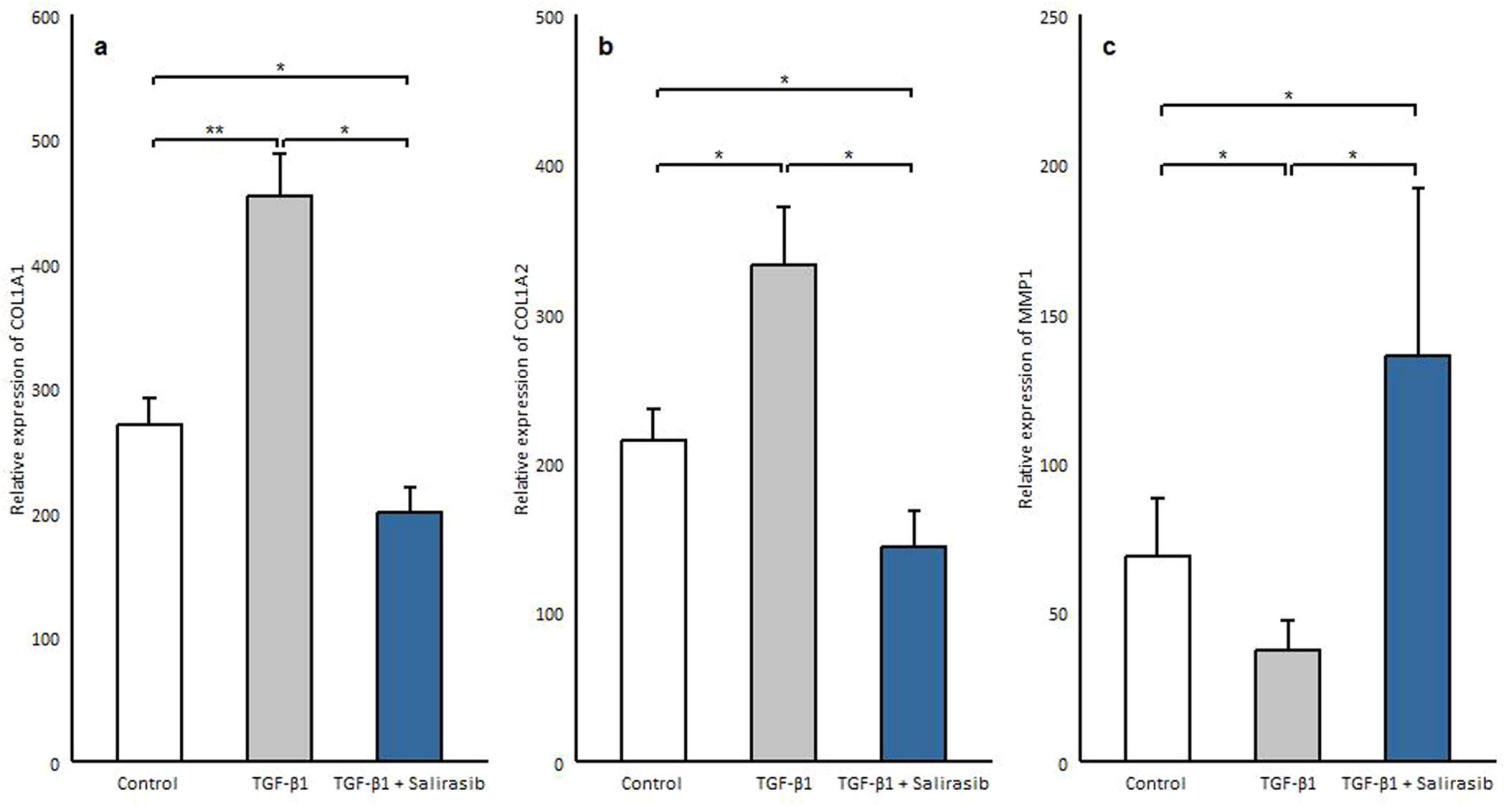
Effects of salirasib on the transcription level of collagen and MMP1 genes in the presence of TGF‐β1. (a, b) The transcription levels of collagen genes and (c) MMP1 was analyzed in the patient's fibroblasts which were treated with 12.5 μm Salirasib and 10 ng/mL TGF‐β1. Values shown are the mean ± SEM. *p < 0.05, **p < 0.01, ***p < 0.001.

MMP‐1 is the main protease that begins to break down the fibrillar collagens type I and III into particular fragments [20]. Considering that TGF‐β1 level correlates with the expression of MMP1 and the degree of collagen deposition [21, 22], fibroblast cells after being cultured in serum‐free medium were treated with 10 ng/mL TGF‐β1 for 72 h, which showed a significant decrease in *MMP1* expression (*p* = 0.018) and increase in the expression of collagen genes (*COL1A1*, *COL1A2*) (*p* = 0.006, *p* = 0.036 respectively) (Figure 5a–c). Then salirasib treatment was done to assess the effect of salirasib on these genes, which showed that salirasib treatment results in a significant decrease in the expression of collagen genes (*COL1A1, COL1A2*) (*p* = 0.012, *p* = 0.024 respectively), and caused a significant enhancement in the mRNA expression of *MMP1*(*p* = 0.012) (Figure 5a–c).

### 3.6 Salirasib Downregulates the Expression of Genes Involved in Fibrosis

**Figure 6:**
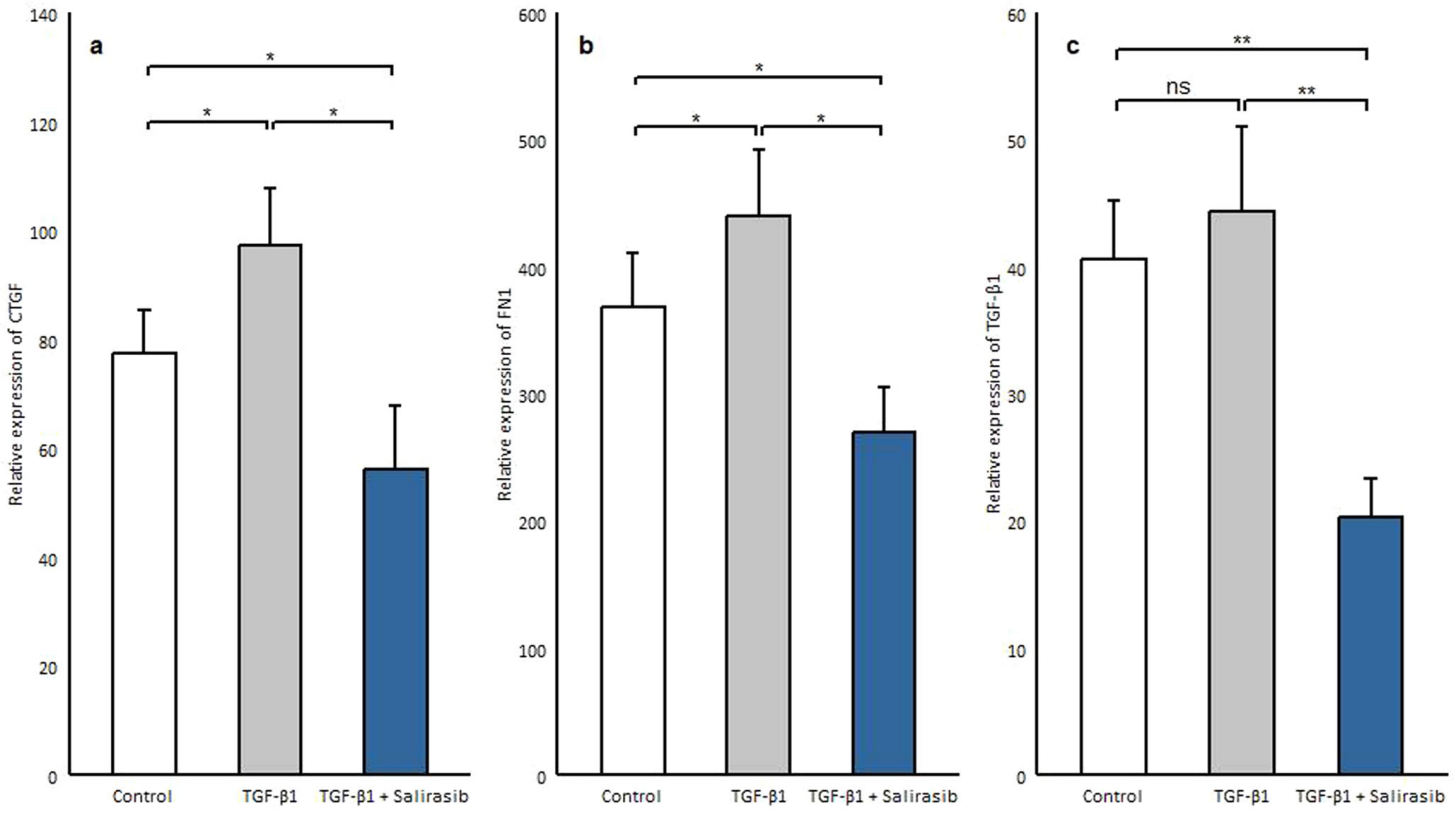
Effects of salirasib on the transcription of fibrosis‐related genes in the presence of TGF‐β1. The mRNA levels of fibrosis‐related genes were evaluated after treatment of the patient's fibroblasts with 12.5 μM salirasib and 10 ng/mL TGF‐β1. Values shown are the mean ± SEM. *p < 0.05, **p < 0.01, ***p < 0.001.

Although TGF‐β is identified as a major trigger in fibrosis, other factors are responsible for maintaining chronic fibrosis such as connective tissue growth factor (CTGF) and FN1 [23, 24]. CTGF as a co‐factor of TGF‐β, contributes to myofibroblast recruitment and secretion of collagen and other extracellular proteins [25, 26]. Fibronectins play an important role in myofibroblast differentiation, collagen production, and augmented matrix stability and collagen cross‐linking in human skin in SSc [27]. To determine the effect of salirasib on the expression of these genes, we treated SSc fibroblasts after serum starvation with TGF‐β1 and salirasib. Our results showed that TGF‐β1 treatment can induce *CTGF* (*p* = 0.012) and *FN1* expression (*p* = 0.012) but, it did not affect *TGF‐β1* gene expression (Figure 6a–c). Besides, the results demonstrated that salirasib reduces the expression of all these three genes significantly (*CTGF, p* = 0.024; *FN1*, *p* = 0.012; *TGF‐β1*, *p* = 0.006) (Figure 6a–c).

## 4 Discussion

Fibrosis is the leading cause of mortality and morbidity in patients with SSc [2]. It distinguishes with the abundant synthesis of ECM components including collagens and fibronectin by fibroblasts and myofibroblasts and inhibition of their degradation via the disruption in the expression or function of MMPs, especially MMP1 [28, 29]. The activation of fibroblasts and myofibroblasts can be regulated by diverse profibrotic cytokines such as TGF‐β [30, 31, 32, 33].

TGF‐β1 is a main profibrotic cytokine that promotes α‐SMA positive myofibroblast development and the enhancement of the expression of several kinds of collagens (I, III, VI, VII, and X), fibronectin and proteoglycans [34, 35]. TGF‐β1 signaling takes place in two distinct pathways, the canonical pathway (Smad‐dependent) and the noncanonical pathway which activate the Ras‐MAPK pathway [10]. Numerous studies have demonstrated that the signaling cascade of Ras‐ERK1/2 can be considered one of the key pathways in the synthesis of ECM proteins and the progress of fibrosis [36, 37, 38, 39, 40]. Different pro‐fibrotic growth factors and cytokines such as ET1, PDGF, IL‐6, and TGF‐β which are increased in skin and serum of SSc patients can enhance ECM production and differentiation of fibroblasts into myofibroblasts through activation of Ras signaling pathway [8, 9]. Furthermore, it has been revealed that the genes associated with the Ras signaling pathway are significantly increased in SSc patients [41] and high expression of these genes was correlated with the disease severity [42]. Our results showed that *H‐Ras* has a significant positive correlation with the main pro‐fibrotic genes including *COL1A2, FN1*, and *TGF‐β* which is consistent with previous studies that imply H‐Ras's role in fibrosis [43].

Salirasib is a derivative of salicylic acid that can reduce all isoforms of the Ras molecule's activity by removing its membrane anchor from the plasma membrane [11], so, salirasib can suppress Ras function. Our results indicated that salirasib significantly decreases the mRNA levels of *H‐Ras* in SSc fibroblasts, which is consistent with previous study showing that salirasib can decrease Ras expression and activity in a dose‐ and time‐dependent manner [44].

Myofibroblasts are activated fibroblast cells and are identified by the overexpression of α‐SMA protein [45]. Our results indicated that treatment of SSc fibroblasts with salirasib decreases the protein expression of α‐SMA and the expression of the *ACTA2* gene. So, it seems that salirasib can reduce the differentiation of myofibroblasts which are the key players in SSc pathogenesis and fibrosis. Furthermore, our study showed that salirasib treatment is related to a significant reduction in the gene expression of *COL1A1* and *COL1A2* that had been augmented by TGF‐β1. Consistent with our result, Yoram Nevo, et al. have shown that salirasib can reduce the fibrosis and inflammation index [46]. In another study, it has been revealed that salirasib can inhibit fibrosis progression and collagen production by suppressing the Ras expression and function and ERK phosphorylation [47]. Concerning collagen as the main composition of ECM, the inhibitory effect of salirasib on the expression of collagen can prevent fibrosis.

MMP1 or collagenase‐1, acts mainly to break down collagen types I and III [4]. Some investigations show significantly reduced expression of the MMP1 gene and protein levels in dermal biopsies of SSc patients. Yuan *et, al*. suggested that TGF‐β1 could prevent the *MMP1* gene expression in fibroblast cells [48]. Interestingly, in our study, the expression of the *MMP1* gene, which was reduced by TGF‐β1, in salirasib‐treated fibroblasts was significantly increased. So, with regard to the impaired ECM degradation in SSc due to defects in MMPs [4], salirasib can lead to the improvement of ECM degradation in fibrotic tissues through MMP1 overexpression.

CTGF acts as a co‐factor of TGF‐β and contributes to myofibroblast recruitment and secretion of collagen and other ECM proteins [49]. CTGF is induced by TGF‐β and in an autocrine loop contribute to the continuous activation of fibroblasts in SSc [50]. Fibronectins are high–molecular weight modular glycoproteins that circulate in soluble form in plasma or assemble in tissue as insoluble ECM components (fnEDA, fnEDB) [51]. The EDA‐containing fibronectin variant (fnEDA) plays a substantial role in myofibroblast differentiation and production of ECM proteins [52, 53]. The results of our study showed that salirasib caused a significant reduction in *TGF‐β*, *CTGF*, and *FN1* gene expression. So, given the effect of salirasib on the expression of these genes that are related to fibrosis development, salirasib can prevent fibrosis.

While our study provides compelling evidence that salirasib inhibits the expression of fibrosis‐related genes in fibroblasts derived from patients with SSc, it is important to acknowledge certain limitations inherent to the examination of protein levels of Ras and genes involved in fibrosis. As this was a pilot study and because of the lack of access to the necessary materials, most of the focus of this study was on the analysis of gene expression, and we did not extend our investigation to the protein level, which is a significant limitation. Protein‐level validation is necessary to confirm that the observed gene expression changes translate into the corresponding protein alterations and functional outcomes. Future studies should incorporate protein‐level analyses to provide a more comprehensive understanding of the molecular mechanisms through which salirasib exerts its effects. Additionally, these studies could explore whether the changes in protein expression align with functional outcomes, such as reduced collagen deposition or improved cellular phenotypes, thereby strengthening the evidence for salirasib as a potential therapeutic agent in SSc.

## 5 Conclusion

Considering our results that showed salirasib can inhibit pro‐fibrotic mediators and myofibroblast differentiation which can result in the prevention of fibrosis, and up‐regulate MMP1 which can lead to the degradation of deposited ECM, it seems that salirasib has the potential to be a new curative strategy for SSc.

## Author Contributions

**Mina Sadeghi Shaker, Mohsen Rokni, Samaneh Enayati,** and **Elham Madreseh:** Acquisition of data, analysis and interpretation of data, drafting of the article. **Hoda Kavosi, Elham Farhadi, Mahdi Mahmoudi,** and **Mohammad Vodjgani:** The conception and design of the study, revising the article critically, interpretation of data, and final approval of the article.

## Ethics Statement

This study was performed based on the Declaration of Helsinki guidelines and‎ was approved by the Ethics Committee of Tehran University of Medical Sciences (Ethics code: IR. TUMS. MEDICINE. REC.1399.1156).

## Conflicts of Interest

The authors declare no conflicts of interest.

## Data Availability

All data generated or analyzed during this study are available upon request.
